# Randomized test-treatment studies with an outlook on adaptive designs

**DOI:** 10.1186/s12874-021-01293-y

**Published:** 2021-06-01

**Authors:** Amra Hot, Patrick M. Bossuyt, Oke Gerke, Simone Wahl, Werner Vach, Antonia Zapf

**Affiliations:** 1 Institute of Medical Biometry and Epidemiology, University Medical Center Hamburg-Eppendorf, Martinistraße 52, Hamburg, 20246 Germany; 2 Department of Epidemiology and Data Science, Amsterdam University Medical Centers, Meibergdreef 9, Amsterdam, 1105 AZ The Netherlands; 3 Department of Nuclear Medicine, Odense University Hospital, J.B. Winsløws Vej 4, Odense C, 5000 Denmark; 4 Department of Clinical Research, University of Southern Denmark, Winsløwparken 19, Odense C, 5000 Denmark; 5 Roche Diagnostics GmbH, Nonnenwald 2, Penzberg, 82377 Germany; 6 Basel Academy for Quality and Research in Medicine, Steinenring 6, Basel, 4051 Switzerland; 7 Department of Environmental Science, University of Basel, Spalenring 145, Basel, 4055 Switzerland

**Keywords:** Accuracy, Adaptive design, Diagnostic research, Patient-relevant outcome, RCT, Sample size, Test-treatment

## Abstract

**Background:**

Diagnostic accuracy studies aim to examine the diagnostic accuracy of a new experimental test, but do not address the actual merit of the resulting diagnostic information to a patient in clinical practice. In order to assess the impact of diagnostic information on subsequent treatment strategies regarding patient-relevant outcomes, randomized test-treatment studies were introduced. Various designs for randomized test-treatment studies, including an evaluation of biomarkers as part of randomized biomarker-guided treatment studies, are suggested in the literature, but the nomenclature is not consistent.

**Methods:**

The aim was to provide a clear description of the different study designs within a pre-specified framework, considering their underlying assumptions, advantages as well as limitations and derivation of effect sizes required for sample size calculations. Furthermore, an outlook on adaptive designs within randomized test-treatment studies is given.

**Results:**

The need to integrate adaptive design procedures in randomized test-treatment studies is apparent. The derivation of effect sizes induces that sample size calculation will always be based on rather vague assumptions resulting in over- or underpowered study results. Therefore, it might be advantageous to conduct a sample size re-estimation based on a nuisance parameter during the ongoing trial.

**Conclusions:**

Due to their increased complexity, compared to common treatment trials, the implementation of randomized test-treatment studies poses practical challenges including a huge uncertainty regarding study parameters like the expected outcome in specific subgroups or disease prevalence which might affect the sample size calculation. Since research on adaptive designs within randomized test-treatment studies is limited so far, further research is recommended.

**Supplementary Information:**

The online version contains supplementary material available at (10.1186/s12874-021-01293-y).

## Background

Diagnostic accuracy studies are performed to assess how well a diagnostic test can distinguish between diseased and non-diseased individuals. Following Schünemann et al.(2008) [1], whenever a diagnostic test fails to improve patient health outcomes, it should not be used in the daily routine, even though it may have proved to be highly accurate. Consequently, the resulting diagnostic information, that is sensitivity and specificity of a diagnostic test, is only beneficial if it is appropriately used in subsequent patient management decisions, and, thus, clinically relevant outcomes, such as morbidity, mortality or health related quality of life, are improved in the long run. For the assessment of a diagnostic test’s efficacy, randomized controlled trials (RCTs) are needed in order to compare test-treatment strategies and to evaluate their performance on patient health outcomes as well as to differentiate effects between patient subgroups or trial arms [2–6]. The focus of randomized test-treatment studies lies in the establishment of a test-treatment process which includes the application of diagnostic test(s) determining a target condition, examining the test results, identifying downstream management strategies through a predetermined link between test results and management decision and finally evaluating its implementation on patient health outcome [2, 6–9]. However, the increased complexity of trial arms employing diagnostic tests as part of the patient management decision process led to diagnostic RCTs taking quite different forms. Various designs for RCTs in diagnostic research have been suggested in the literature, but the nomenclature is not consistent [8–12].

The development of molecular and genetic technologies has strengthened the understanding of the molecular structure of a disease, which increased the value of biomarkers for personalized medicine [13–17]. Biomarkers are characteristics that can be used to indicate normal and abnormal biological mechanisms or predict a patient’s response to a therapeutic intervention [13, 18]. The clinical value of biomarkers for patient-related outcomes is demonstrated in biomarker-guided treatment studies, which are planned to evaluate prespecified biomarker-based treatment strategies [13, 14, 16, 17, 19–21]. Here, biomarkers can be classified in prognostic and predictive biomarkers that are relevant for the selection of individualized treatment strategies [19]. If randomized biomarker-guided treatment studies compare a biomarker-based strategy for treatment allocation with another strategy, they provide a special case of a randomized test-treatment study. For example, eligible patients are randomized to a biomarker-based treatment strategy or a control arm without biomarker evaluation. Both aim to assess the efficacy of management strategies in test- or biomarker guided subgroups of patients and evaluate patient-related outcomes. Several types of biomarker-guided treatment studies, including the treatment-by-marker interaction designs, have been introduced in the literature. Similarly, as in diagnostic RCTs without biomarker evaluation, the nomenclature in biomarker-guided treatment studies is not consistent. Tajik et al.(2013) [21] offer an overview of different designs in the context of biomarker-guided treatment studies. However, for the remainder of this article, we reckon that the term “randomized test-treatment studies” covers all designs which aim to evaluate test and treatment strategies together regarding patient-relevant outcomes, regardless of the type of biomarkers or diagnostic tests used. In the context of this paper, the focus is to evaluate the effect of a diagnostic test in terms of the impact on patient outcome that is led by the test results, rather than treatment selection. Test assessment therefore requires the consideration of all links included in the entire test-treatment-process.

An essential part of planning a clinical trial is the sample size calculation. The study should be adequately powered to detect a potential test-treatment effect. However, at the planning stage the sample size calculation of randomized test-treatment studies is based on assumptions regarding the difference in treatment effects resulting from the test-based strategies, prevalence of disease and diagnostic accuracy of the investigated tests. Due to possibly incorrect assumptions, there is always a risk of over- or underestimation of the sample size. An adaptation of the initial sample size would be desirable to achieve a sufficient, but not overpowered study. For example, a sample size re-estimation based on the prevalence or diagnostic performance would be possible, if a reference standard is applied in a blinded manner in addition to the tests to be compared.

The main contribution of this paper is to present a structured overview of existing randomized test-treatment studies. In doing so, we want to emphasize the complexity of such studies and, as a result, provide a first insight into the potential of adaptive designs, especially sample size re-estimation. The aim of this article is not to present a comprehensive adaptive design in the context of such studies. This aspect, and in particular the extent to which the sample size can be adjusted and what assumptions have to be made, is part of further research.

The paper is organized as follows: we start with some general notations which apply to all presented designs. Afterwards, every design is described systematically within a pre-specified framework: firstly, we point out some basic principles of using testing procedures to individualize treatment and describe their purpose as part of RCTs, including the hypotheses to be tested. Further, for each design, a derivation of the effect size required for sample size calculations is provided. Thereafter, an outlook on adaptive designs within randomized test-treatment studies is given. Each design as well as a potential adaptive design is illustrated by an example study. In the last section, we close with a brief discussion of the findings. Formulas for sample size calculation for binary and continuous outcomes are given in the Appendix (see Additional File 1).

## Methods

In the following, three main designs are described: the classical test-treatment RCT, the design with restriction to discordant pairs, and the design with random disclosure. The description follows a unified framework consisting of seven aspects: (1) motivation, (2) aim, (3) basic set-up, (4) hypotheses to be tested, (5) limitations, (6) nomenclature and (7) example studies.

### General notation

At the beginning of this section we provide some general notation applying to all designs considered. Firstly, let *D*∈{+,−} be the true disease status of the individuals, where *D*=+ denotes the truly diseased and *D*=− the non-diseased state. Hence, *P*(*D*=+) refers to disease prevalence of the population. It is a common property of all designs to compare test-treatment strategies that in general involve the application of two (diagnostic) tests with binary response. Let $\mathcal {T} \in \{A,B\} $ denote the test applied to a patient and $R_{\mathcal {T}} \in \{\text {+},\text {--}\}$ the result of the corresponding test $\mathcal {T}$. In the following we always assume that an experimental test *A* is compared with a comparator test *B*. It may also be the case that a test or biomarker based strategy is compared to a non-test or non-biomarker based strategy, especially in the absence of a reference standard, in order to compare it with the test under investigation or when the predictive value of a biomarker is to be assessed [13, 15, 21]. Let $\mathcal {M} \in \{\mathrm {I},\text {II}\}$ denote the intervention (management strategy) given to the patient. Management strategy I may be a more invasive treatment or therapeutic approach which should work better for truly diseased patients and management II may represent a standard of care which should work better in truly non-diseased patients. It is essential that these tests and their application as well as management strategies are clearly pre-specified in the study protocol. A well-defined linking rule of the test results and subsequent management decisions is mandatory to assure validity and generalizability of the study results [2, 3, 6, 7, 9]. Finally, *Y* defines a binary or continuous patient outcome which is measured after receiving a treatment strategy.

### Classical test-treatment RCT

*Motivation*: When a new experimental test has shown improved accuracy, it can still be unclear whether its application implies a benefit for the patients in the long run, as this requires translating the improved test results into improved treatment and consequently improved outcomes in clinical practice. Hence, there is a need to examine whether the application of the test indeed improves long-term outcomes. For this purpose, classical RCTs in diagnostic research are introduced.

*Aim*: The aim is to compare two diagnostic procedures with respect to their impact on patient-related outcomes. Classical RCTs evaluate the application of the diagnostic tests together with a treatment strategy (or more generally a management strategy) based on the test results. This strategy can range from a fixed, well-defined algorithm to a very liberal approach only requiring to take the results into account in the (complex) management of patients.

**Fig. 1:**
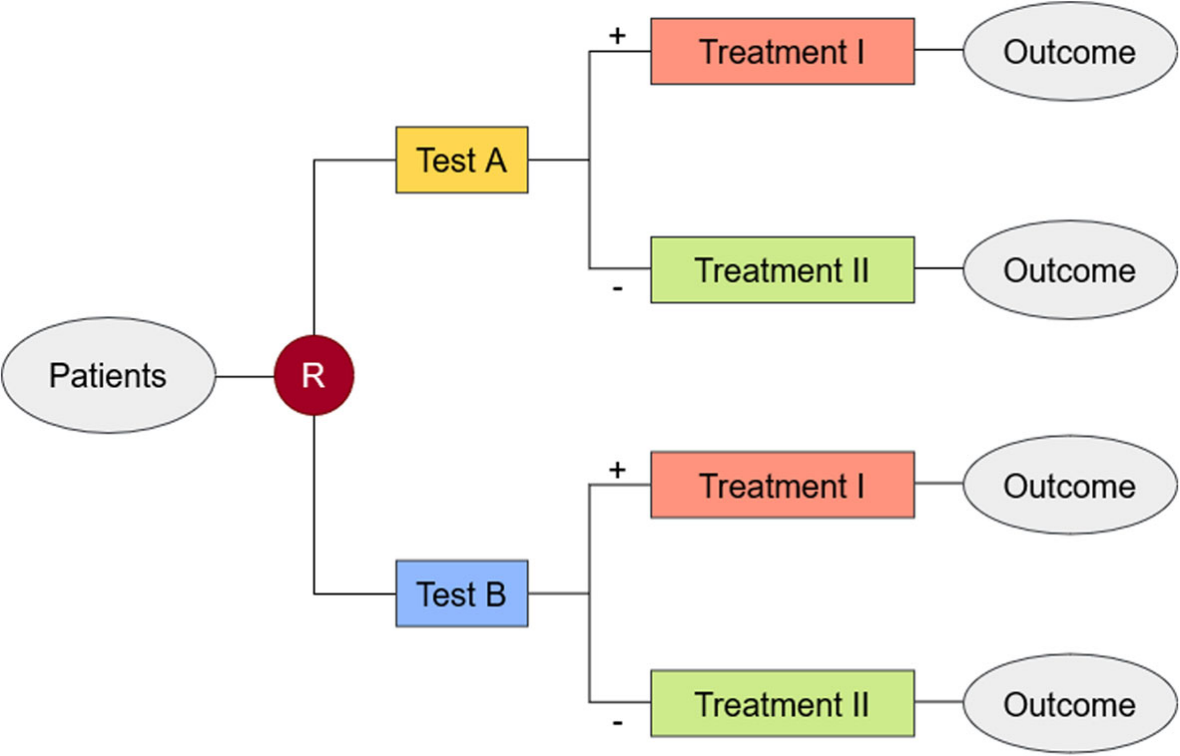
A schematic representation of a classical test-treatment RCT [9]

*Basic set-up*: Patients are randomized to two arms. In one arm, the binary test *A* is applied, in the other, *B*. Test results are communicated and associated with subsequent management decision: test-positives are assigned to treatment I and test-negatives to treatment II. Afterwards, the patient outcome is evaluated in each subgroup (Fig. 1).

*Hypotheses*: The hypothesis of interest in the classical RCT design refers to test whether there is a difference in outcome between the test-treatment path based on test *A* compared to the test-treatment path based on test *B*. The hypothesis can be formulated as

$$\mathrm{H}_{0}: \theta_{A} = \theta_{B} \hspace{15pt} \text{vs.} \hspace{15pt} \mathrm{H}_{1}: \theta_{A} \neq \theta_{B}, \hspace{10pt} $$

with $ \theta _{\mathcal {T}} := E(Y | \mathcal {T} = \tau)$ denoting the expected outcome in each arm *τ*=*A*,*B*. Sample size calculations hence require qualified guesses for *θ*_*A*_ and *θ*_*B*_, or at least for the difference between these two numbers. Two different approaches to arrive at this information are outlined in the next section. If such qualified guesses are given, sample size calculations can follow traditional routes as shown in the Appendix (see Additional File 1).

*Limitations*: In case of negative study results, it is unknown whether this is due to an insufficient difference in accuracy between *A* and *B*, suboptimal use of the test results in further treatment decisions, or due to low effectiveness of subsequent treatments, as the effect of entire test-treatment strategies is assessed. None of the patients received both test procedures, thus, it is not possible to distinguish the treatment effect from the prognostic or predictive value of the tests, nor is it possible to compare the outcome in the subgroups with discordant test results. In general, blinding of the physician or patient to the test results and thus treatment allocation is not possible, as there is a disclosure of the test results and treatment assignment [9]. Blinding regarding the applied testing procedures is possible only if testing procedures are based on similar approaches, e.g. magnetic resonance tomography for the diagnosis of one disease based on different parameters which are compared to each other. Further, the sample sizes required for this design are usually large, as enough patients are needed in both testing arms to detect a difference between the entire test-treatment pathways.

*Nomenclature*: Such studies have been called classical RCT [13], RCT comparing tests [8], ungated RCT [22] and two-arm design [10]. If a test-based strategy and the standard, non-test-based strategy are compared, this design has been called test RCT [8] and due to its prominent role in biomarker research, biomarker-strategy design [14], marker-based strategy design I [15, 16, 23], biomarker-strategy design with standard control [20], and marker strategy design [17].

*Example*: A randomized diagnostic study used this design to investigate two different diagnostic approaches for the management of outpatients with dysphagia, who have a high risk for developing aspiration pneumonia [24]. In this prospective, randomized study it was investigated whether a modified barium swallow test (MBS), test A, and flexible endoscopic evaluation of swallowing with sensory testing (FEESST), test B, as diagnostic tests are supposed to distinguish patients who can benefit from behavioral and dietary management (treatment II) from those who will need a percutaneous endoscopic gastrostomy (PEG) tube (treatment I). The outcome variables were pneumonia incidence and pneumonia-free interval. In total, 126 outpatients with dysphagia were randomized to either FEESST or MBS.

### Restricting randomization to discordant pairs

*Motivation*: The limited power of classical RCTs in diagnostic research is partially due to the fact that in many patients the results of the two tests are identical, and consequently the management will be the same. Only in patients with discordant results we can expect a difference in management and hence a difference in outcome (see classical RCT). This has led to the idea to restrict randomization to patients with discordant results [9].

*Aim*: Both tests *A* and *B* are applied in all patients. This makes it possible to assess individual effects of the tests and the strategy difference in cases with discordant test results. We can compare whether it is preferable to follow the results of the experimental test *A* or the comparator test *B* in discordant cases.

**Fig. 2:**
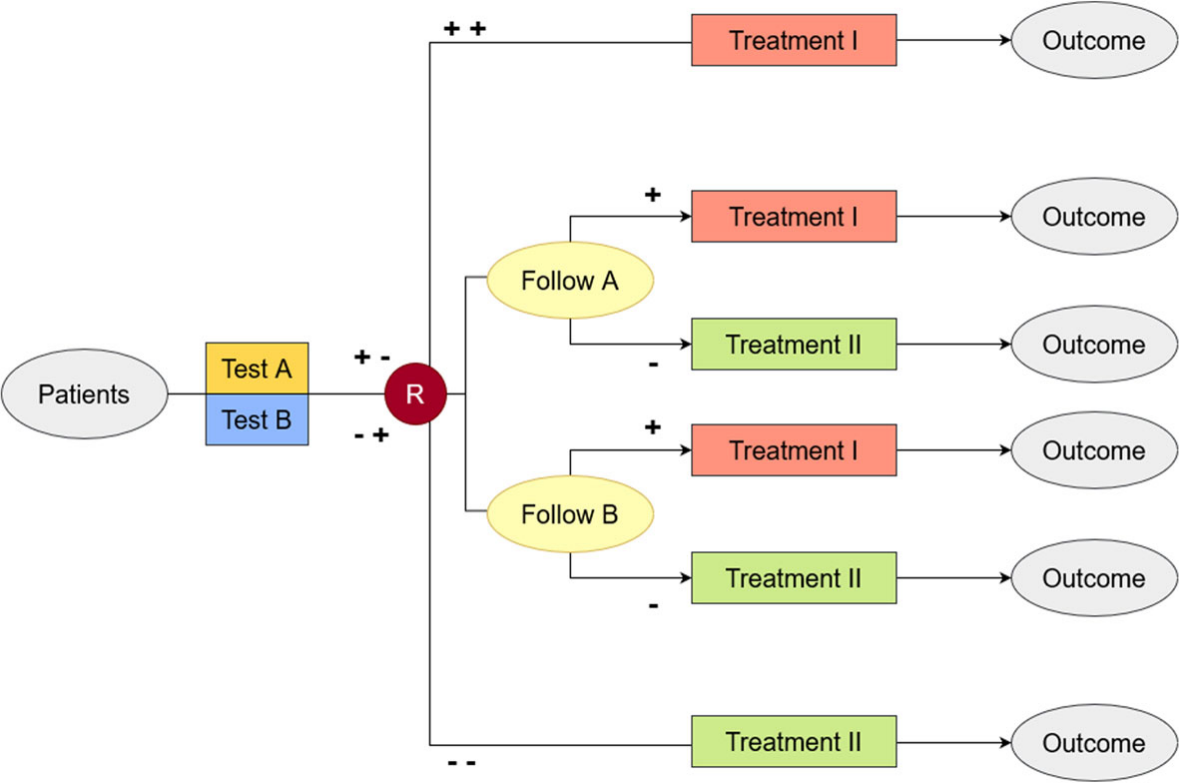
A schematic representation of a randomized diagnostic study with restricting randomization to discordant pairs [9, 25]

*Basic set-up*: After application of both tests patients with discordant test results are randomized to follow management based on test *A* or *B*. Concordant cases with positive test results receive treatment I and test-negative patients receive treatment II (Fig. 2).

*Hypotheses*: The hypotheses of interest for the discordance design can be stated as

$$\mathrm{H}_{0}: \theta^{disc}_{A} = \theta^{disc}_{B} \hspace{15pt} \text{vs.} \hspace{15pt} \mathrm{H}_{1}: \theta^{disc}_{A} \neq \theta^{disc}_{B} $$

with

$$\theta^{disc}_{\tau} = E \left(Y | \mathcal{T} = \tau, R_{A} \neq R_{B} \right) $$

and $\mathcal {T}$ now denoting the test to be followed according to the randomization. Again, qualified guesses are required for these numbers or at least for the difference. We discuss in the next section corresponding approaches, which are similar to those for classical test-treatment studies.

*Limitations*: Similar to the classical design blinding of the physician and patient to the test result and treatment allocation is difficult. In discordant cases, results of tests *A* and *B* cannot be deduced and are, therefore, blinded, whereas the concordance or discordance of test results cannot be kept secret. Hence, while the design has the advantage of greater efficiency [10], the question remains, whether the management of patients can be influenced by the fact that the treating clinicians are aware of treating a patient with discordant test results, and to which degree results obtained from this design are indeed comparable to results from the classical design. In contrast, a study in discordant pairs allows also to investigate treatment effects separately in the two different types of discordant pairs. It might be the case that it is wise to follow the new test, if it changes a positive standard test to a negative result, but not vice versa. Designs where each patient undergoes both tests are only feasible, if both tests are applicable in each patient and the performance of the tests is not affected by each other [9]. For example, this may not be the case with surgical testing procedures. Additionally, both tests should deliver test results within comparable timeframes as different ones might influence the study results [2].

*Nomenclature*: This design has been called gated RCT [22], discordant risk randomization design [20], RCT of discordant test results [8], paired design [10], and marker discordance design [17].

*Example*: An example of a trial in which patients with discordant test results are randomized is the FOAM study, which compares the cost-effectiveness of hysterosalpingo-foam sonography (HyFoSy) with hysterosalpingography (HSG) in assessing tubal patency in subfertile women [26]. The study is planned as a multicenter prospective study of women undergoing tubal patency testing by HyFoSy (test *A*) and HSG (test *B*) during fertility work-up. Women in this study with discordant test results are randomized to a management strategy based on HyFOSy (management strategy I) or HSG (management strategy II) resulting in a diagnostic laparoscopy with chromopertubation or a management based on the prognostic model of Hunault. Data are used in a model-based cost-effectiveness analysis. The primary endpoint is pregnancy rates within 12 month after inclusion. In total, 1,163 subfertile women between 18 and 41 years of age who are scheduled for tubal patency testing during their fertility work-up will be enrolled to the study.

### Random disclosure

*Motivation*: A hybrid between a full classical randomized test-treatment study and a study in discordant pairs arises if both tests are applied in all patients, but randomly only one test result is disclosed and the other is kept secret. This design implements the random disclosure principle [9]. It has the advantage that it can be analyzed like a randomized study, but additionally and timely subsequently also as a study in discordant pairs.

*Aim*: The aim of this study design is to test whether a strategy based on a test *A* with improved accuracy should be preferred to a test-treatment strategy based on test *B* for all patients recruited to trial. Furthermore, it is possible to compare the tests in the patients with discordant test results.

**Fig. 3:**
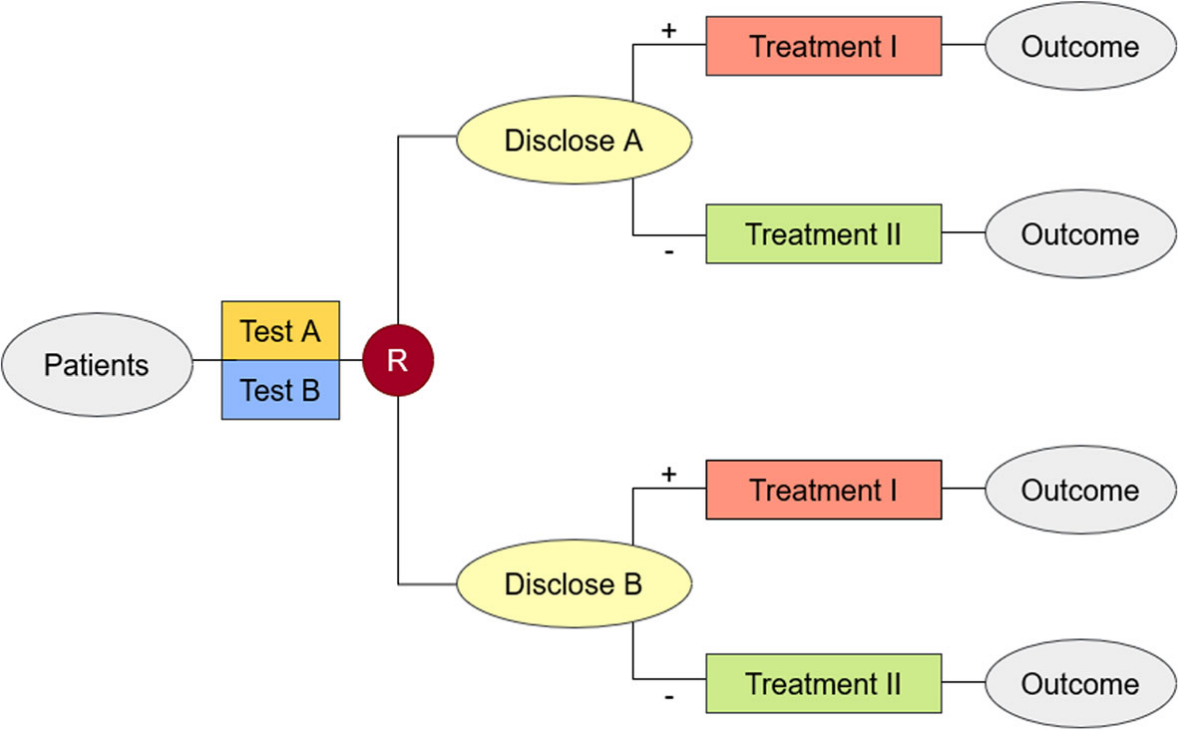
A schematic representation of a study design with random disclosure principle [9]

*Basic set-up*: Two tests *A* and *B* are applied in all patients, which are randomized to two arms, in each of which only the result of one test is revealed. Subsequently, test-positive and -negative patients are assigned to treatment I and II, respectively (Fig. 3).

*Hypotheses*: Data from a study using a random disclosure design can be analyzed like a classical RCT or like an RCT in discordant pairs, since on the one hand the aim is to compare two entire test-treatment paths, on the other hand we can, additionally, perform a subgroup analysis in the discordant cases. Sample size calculations can be performed with respect to one of the two possible analytic strategies. Hence the considerations presented for the two other designs can be also applied here.

**Fig. 4:**
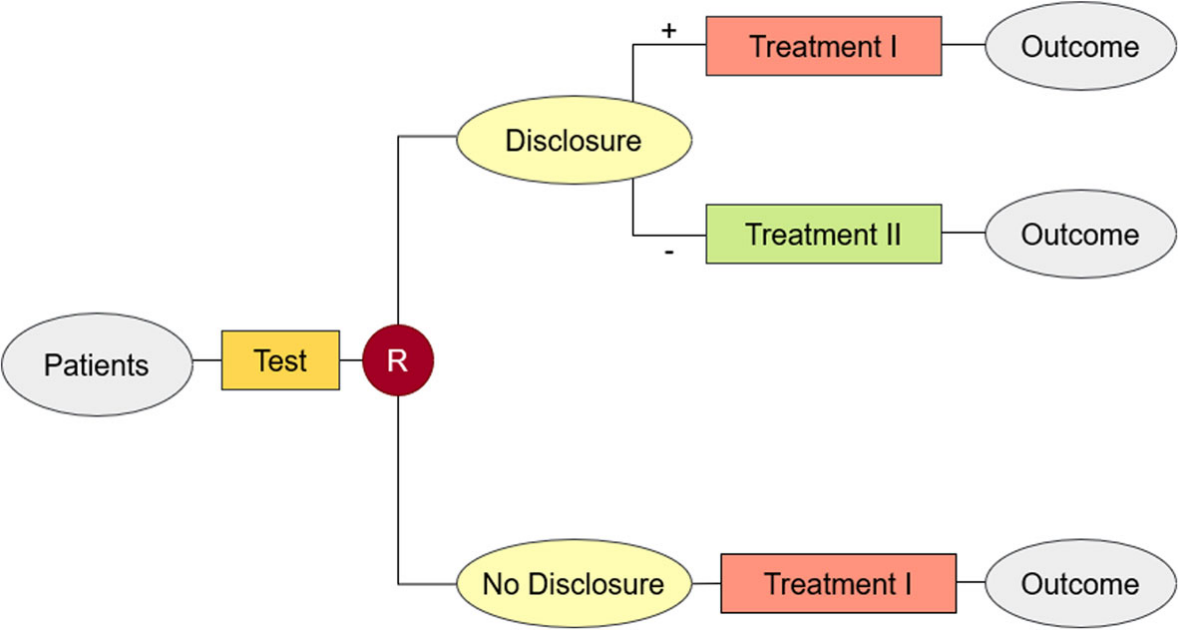
A schematic representation of a study design with random disclosure of one diagnostic test [9]

*Variant*: This design can be reduced to considering only one test. Here, all patients are randomized to either a disclosure or non-disclosure of the test results. Patients with a positive or non-disclosed test result receive treatment I, whereas patients with a negative test result are allocated to treatment II (Fig. 4). Here, treatment I is considered to be the therapy patients would receive for a specific disease. This additional arm can provide information about the prognostic value of the test, as it is often the case in biomarker studies.

*Limitations*: This design is only applicable if it is feasible to apply both tests in all patients. Similar to the classical RCT design, in general, blinding of the physician and patient regarding the test results and treatment allocation is difficult, as test-positive patients receive treatment I and test-negative treatment II. In case that the applied tests are based on different methods, e.g. a biochemical test is compared to an imaging technique, blinding regarding the applied tests is possible.

*Nomenclature*: This design is called study regarding the random disclosure principle [9] or random disclosure design [11] in case of considering one diagnostic test.

*Example*: In a randomized study, this design was used to evaluate Doppler ultrasonography (Doppler US) (diagnostic test) of the umbilical artery in the management of women with suspected intrauterine growth retardation (IUGR). In total, 150 women with singleton pregnancies and suspected IUGR were randomized to an intervention (disclosure group) and a control group (non-disclosure group). In the intervention group, clinicians were strongly requested not to hospitalize for suspected IUGR if the Doppler US findings were normal. In the control group, the Doppler US results were not revealed and the participants received the standard management for suspected IUGR. Endpoints of the trial were costs in terms of hospitalization, perinatal outcome, neurological development and postnatal growth [27].

## Sample size considerations

In the following section, sample size considerations are presented that start with two different perspectives. The first perspective refers directly to an unpaired study design in which study participants are assigned to one of the two testing procedures. This approach directly aims at deriving formulas for the expected outcome in each arm. The second perspective takes into account from the start that we have discordant and concordant test results and aims at the difference in expected outcomes. At first sight this may be seen to be relevant only for studies in discordant pairs. However, this perspective is also relevant for planning a test-treatment RCT, as the information we need can be derived from paired accuracy studies and does not require a paired design of the RCT.

### Unpaired approach

For a sample size calculation in the classical RCT design, we can try to derive at a qualified guess for the expected outcome $\theta _{\mathcal {T}}$ for $\mathcal {T} = A,B$. If we assume that the test result determines the choice of treatment, i.e. that $\mathcal {M} = m(R_{\mathcal {T}})$ with *m*(+)=I and *m*(–)=II, then we can express the expected outcomes for test $\mathcal {T}$ as

$$\begin{aligned} \theta_{\tau} &= \sum_{t \in \{\text{+},\text{--}\}, d\in \{\text{+},\text{--}\}} \mu^{\tau}_{m(R_{\tau})td} P\left(R_{\tau} = t | D=d, \mathcal{T} = \tau\right) P\left(D=d | \mathcal{T} = \tau\right) \\ &= \sum_{t \in \{\text{+},\text{--}\}, d\in \{\text{+},\text{--}\}} \mu^{\tau}_{m(R_{\tau})td} P(R_{\tau} = t | D=d) P(D=d) \end{aligned} $$

with

$$\mu^{\tau}_{mtd} = E \left(Y | \mathcal{M}=m, R_{\tau} = t, D=d\right). $$

To use this expression for a sample size calculation, we have to discuss how to determine the different components. The parameter *P*(*D*=*d*) is known if we can make a reasonable assumption about the disease prevalence in the study population. *P*(*R*_*τ*_=*t*|*D*=*d*) refers to the sensitivity and specificity of the two tests, i.e. the proportion of truly diseased and non-diseased patients, respectively, who are correctly identified [28]. This may be known from accuracy studies. The crucial part is, however, to arrive at assumptions about $\mu ^{\tau }_{mtd}$. We cannot make the simplifying assumption that $\mu ^{\tau }_{mtd}$ is independent of *t*∈{+,−}, as a positive or negative test result can have a prognostic value on top of the true disease state (e.g. it might be related to disease progression). We can neither assume that $\mu ^{\tau }_{mtd}$ is independent of *τ*∈{*A*,*B*}, as the prognostic value of the two tests may differ, nor we can expect to be able to estimate $\mu ^{\tau }_{mtd}$ empirically from existing studies for all combinations of *τ*, *m*, *t*, and *d*. Typically, I is intended for patients with *D*=+ and II for patients with *D*=–, hence it is often unlikely to find studies looking at the opposite combinations [22]. Moreover, if *A* represents a new test, it is also unlikely to find outcome studies using *A* to determine the disease status. This challenge will be discussed in the last section.

The sample size *n* needed for this trial design can be calculated by inserting the estimates of *θ*_*A*_ and *θ*_*B*_ in the sample size formulas in the Appendix (see Additional File 1).

### Paired approach

For a sample size calculation it might be sufficient to have a qualified guess for *Δ*=*θ*_*A*_−*θ*_*B*_. An alternative expression for this can be derived based on differentiating between concordant and discordant pairs, which may also give a better insight how effects of the interventions I and II interfere with the outcome difference we can expect. This requires the application of both tests in all patients, which corresponds to a paired design, as it is the case in the discordance or random disclosure design. In particular we have:

$$\begin{array}{@{}rcl@{}} \Delta \!& =\! & \theta_{A} - \theta_{B} \\ \!& =\! & \sum_{t_{A}, t_{B}, d \in \{\text{+},\text{--}\}} \Delta^{\tau}_{t_{A}t_{B}d} P\left(R_{A} \,=\, t_{A}, R_{B} \,=\, t_{B} | D=d \right) P(D=d) \\ \end{array} $$

with

$$\begin{aligned} \Delta^{\tau}_{t_{A}t_{B}d} &= \phantom{-} E\left(Y | \mathcal{T}=A, \mathcal{M}=m(R_{A}), R_{A}=t_{A}, R_{B}=t_{B}, D=d\right) \\ & - E\left(Y | \mathcal{T}=B, \mathcal{M}=m(R_{B}), R_{A}=t_{A}, R_{B}=t_{B}, D=d\right). \end{aligned} $$

Now it is rather safe to assume that *R*_*A*_=*R*_*B*_, i.e. concordant test results, imply $ \Delta ^{\tau }_{t_{A}t_{B}d} =0$ for any test result and disease status: if both tests give the same result, they should imply the same intervention. So from the eight summands above only four remain. Moreover, each summand can be associated with a clinically relevant situation with respect to exchanging test *A* with test *B* in a single patient. We can move from a false positive (FP) to a true negative (TN) decision or vice versa, or we can move from a false negative (FN) to a true positive (TP) decision (or vice versa). If we denote with *P*(·) the probability to observe a patient with a specific move, and with *Δ*(·) the expected difference in outcomes in such a patient, we can simply express the expected outcome difference in the trial [29] as

$$\begin{aligned} \Delta &= \phantom{+} \Delta (\text{FP} \rightarrow \text{TN}) P(\text{FP} \rightarrow \text{TN}) + \Delta (\text{TN} \rightarrow \text{FP}) P(\text{TN} \rightarrow \text{FP}) \\ & + \Delta (\text{FN} \rightarrow \text{TP}) P(\text{FN} \rightarrow \text{TP}) + \Delta (\text{TP} \rightarrow \text{FN}) P(\text{TP} \rightarrow \text{FN}). \end{aligned} $$

This expression corresponds nicely to an intuitive expectation about the outcome difference: it depends on how many patients will move from incorrect to correct decisions (or vice versa) and how big the gain (or loss) in expected outcome is in these patients. From the perspective of sample size calculation it requires to know the four probabilities and the four expected outcome differences. Estimates of the four probabilities can be derived from accuracy studies comparing the tests *A* and *B* in a paired design. If such a study is not available, they can be based on prior knowledge of sensitivity and specificity of the two tests combined with assumptions about the degree of concordance in diseased or disease-free patients. It is usually hard to imagine finding empirical estimates for the four *Δ* values involved, as they would require outcome studies in discordant pairs. However, it might be feasible to elicit expectations about these values from clinicians. Typically, it will suffice to elicit two values, as the assumptions *Δ*(FP→TN)=−*Δ*(TN→FP) and *Δ*(FN→TP)=−*Δ*(TP→FN) are rather safe. In the case of a continuous outcome, we have to supplement a guess about *Δ* with a guess about the variation of *Y*. Note that within this approach we do not need to assume explicitly that the treatment choice is uniquely determined by the test results.

With respect to the difference in expected outcome $\Delta ^{disc} := \theta ^{disc}_{A} - \theta ^{disc}_{B}$ we have

$$\begin{aligned} \Delta^{disc} & = \sum_{\substack{ t_{A}, t_{B}, d \in \{\text{+},\text{--}\},\\ t_{A} \neq t_{B}}} \Delta_{t_{A}t_{B}d} P\left(R_{A} = t_{A}, R_{B} = t_{B}| D=d, R_{A} \neq R_{B} \right)\\ &\quad P\left(D=d|R_{A} \neq R_{B}\right) \\ & = \frac{\sum_{t_{A}, t_{B}, d \in \{\text{+},\text{--}\}, t_{A} \neq t_{B}} \Delta_{t_{A}t_{B}d} P\left(R_{A} = t_{A}, R_{B} = t_{B}| D=d\right) P(D=d)}{ P\left(R_{A} \neq R_{B} \right)} \\ & = \frac{\Delta} {P\left(T_{A} \neq T_{B} \right)}. \end{aligned} $$

Here, *P*(*R*_*A*_≠*R*_*B*_) refers to the fraction of discordant test results in the population of interest. A sample size determination hence requires specifying exactly the same quantities as in the case of a classical RCT and additionally needs assumptions about the discordance fraction of test *A* and *B*. The number of discordant cases *n*^*disc*^ needed for this trial design can be calculated by inserting *Δ*_*disc*_ in the sample size formula in the Appendix (see Additional File 1). The required total sample size of concordant and discordant cases can be determined by dividing *n*^*disc*^ by the discordance fraction. Lu and Gatsonis (2013) [10] proposed a similar approach for sample size calculation in randomized test-treatment studies, including the classical RCT and the design with restriction to discordant pairs, and discussed a strategy to determine the discordance fraction. In addition, numerical examples were introduced to show the difference in the sample size between the two study designs.

In practice, in most cases diagnostic tests are highly conditionally dependent on the disease status of the patient, i.e. the response of one test changes the probability of response of the other test [30–32]. For example, in case of positive dependence of two tests a patient with a positive test result on the one test will rather be tested positively on the other test and vice versa. Thus maximally negative dependence between two tests will maximize the number of discordant cases, whereas maximally positive dependence results in a maximal number of concordant cases [32]. For sample size determination assumptions about the degree of conditional dependence and hence concordance of the two tests are required.

## Outlook on adaptive designs

Adaptive study designs with group sequential designs as special case allow pre-planned interim analyses, which may lead to an early stopping of the study (for futility or efficacy) or to modifications of design aspects, including sample size [33]. Such adaptive study designs are well established in intervention trials, but are much less common in diagnostic trials [34], especially in randomized test-treatment studies. Already in diagnostic accuracy studies there may be a need for flexible designs, for example to take into account that a presumed prevalence could not be reached. At first glance RCTs in diagnostic research seem to be comparable to RCTs in therapeutic research, but at second glance differences become obvious. In this work, for each design formulas for the effect size are derived, which can inform a sample size determination. It is a specific feature of all these formulas that they combine information on the disease prevalence and accuracy of the diagnostic tests with assumptions on the expected outcome or outcome difference in subgroups defined by disease state and test results. Empirical information on the former may be available from corresponding studies, but empirical information on the latter is typically missing, and we have to work with some qualified guesses. Hence sample size considerations will always be based on a rather weak foundation, making it advisable to check the assumptions about the group differences early in these studies and to allow sample size adaptations. In principle, corresponding adaptive designs can make use of estimates of the group difference as in any intervention trial. However, we can also make use of the original formulas to derive the sample sizes and try to update the pieces of information used in these formulas. For example, we can also apply the reference test in a blinded manner in these designs, allowing to correct for the estimates of prevalence and diagnostic accuracy. Alternatively we may incorporate information from new accuracy studies. We may also update information on expected outcomes or outcome differences in certain subgroups which we can derive from other ongoing studies. Since the small group of patients with discordant results is driving the intervention difference, it is of specific interest to obtain external information for this subgroup. This requires access to studies applying both tests in all patients. It is an attractive feature of the random disclosure design that such additional information can be generated already within the study of interest. In summary, there are different options to implement adaptive designs for sample size recalculation in randomized diagnostic studies. Research is needed to identify the optimal approaches. However, in the following we will illustrate the potential of adaptive designs using a practical example.

In the previous section describing the classical RCT design a randomized diagnostic study was introduced, which used the classical design to investigate which of two diagnostic approaches, the MBS or FEESST, is more effective in managing patients with dysphagia with respect to pneumonia incidence [24]. In total, 126 outpatients with dysphagia were randomized to either FEESST or MBS. Out of 76 patients examined by the MBS, 14 patients (18.4%) developed pneumonia compared to 6 patients out of 50 (12%) in the FEESST group resulting in a statistically non-significant difference between the test-treatment arms. This result can be attributed to the relatively low prevalence in each diagnostic group. A description of the initial sample size calculation of the study is not sufficiently provided by the authors, therefore, we base a re-estimation of the needed sample size on the observed study results, i.e. the observed pneumonia rates. To detect a statistically significant difference of 6.4% with a power of 80%, one would need a total of 986 patients in the trial (based on the Chi-squared test for two independent proportions). In this study, the authors could have used the following adaptive design: first, an initial sample size planning would be made based on assumptions regarding the diagnostic accuracy of the tests, the prevalence of the disease and the treatment effects in the individual subgroups. After a certain number of patients has been recruited, say 50% of all originally intended, a sample size re-estimation based on the prevalence could be performed, as long as the reference standard has been included. At this point, it might have been clear that additional patients would need to be recruited, or that the needed sample size is not feasible and the trial is stopped early for futility. The previously outlined procedure describes a blinded re-estimation of the sample size based on the prevalence, so that the type I error is not inflated. A re-estimation of the sample size based on the sensitivity and specificity or the different treatment effects is expected to lead to an unblinding of the test-treatment allocation, resulting in an unblinded interim analysis. An interim analysis based on the primary outcome (e.g. mortality) would obviously implicate unblinding. This would then necessitate an adjustment of the type I error rate.

Until now, to the best of our knowledge, there is little research regarding adaptive designs for RCTs in diagnostic research. A regulatory guidance on adaptive designs for medical device clinical studies became effective quite recently [33]. We found a few clinical examples using adaptive designs in biomarker research. Heckman-Stoddard and Smith (2014) [35] discussed two adaptive clinical trials programs (I-SPY and BATTLE on breast and lung cancer, respectively) which comprised several innovative randomized studies designed to evaluate multiple targeted therapies in biomarker-defined subsets of individuals. Allison [36] remarked the potential of biomarker-led adaptive trials in breast cancer. Corey et al. [37] reviewed narratively the status of human immunodeficiency virus (HIV) vaccines and discussed the potential role of adaptive clinical trial designs in accelerating vaccine development; as the lack of a predictive animal model and undefined biomarkers of immune protection against HIV necessitate testing of potentially promising vaccines directly in clinical trials. Zhang et al. [38] investigated optimal biomarker-integrated adaptive trial designs, described the performance of the optimal design in different scenarios, and compared it to Bayesian adaptive randomization. Finally, Antoniou et al. [39] performed a methodological review on biomarker-guided adaptive trial designs in phase II and phase III over one decade. They identified eight distinct biomarker-guided adaptive designs and nine variations from 107 studies, and they observed substantial variability in description and terminology. Placzek and Friede [40] considered designs with multiple nested subgroups and a continuous endpoint and developed methods for the analysis and sample size determination, including a blinded sample size re-estimation procedure in an internal pilot study. Gao et al. (2016) [41] proposed a two-stage adaptive design that provides flexibility in a single biomarker performance-based sample size adaption for targeted trials, in which biomarker-positive patients are randomized to a novel treatment of interest or control. Future work is needed to integrate adaptive design procedures in diagnostic RCTs comparing test-treatment strategies with a stronger focus than before on sample size calculation and re-estimation based on a nuisance parameter.

## Discussion and conclusions

Patient health should be the primary concern in evaluating diagnostic tests after sufficient accuracy has been proven. In clinical practice, there are often multiple tests which have the aim to determine the disease status of a patient. When two competing diagnostic tests are compared, randomized test-treatment studies are needed to evaluate the clinical utility of the tests as part of a broader management regimen. In instances where no reference standard is available at all or the reference standard is assumed to be imperfect, diagnostic RCTs may be even the only way to assess the clinical value of a diagnostic test. Several designs of randomized test-treatment studies have been suggested earlier. Depending on its clinical research question, the feasibility and its advantages and limitations, one design can be preferred over the other. The main difference of the designs is the timepoint of randomization, hence leading to slightly different research questions. In the classical diagnostic RCT, fewer tests are, on average, performed than in the remaining designs, as each patient receives only one test. This leads to a less cost-intensive and time-consuming design in case of expensive and laborious testing procedures [9]. Furthermore, in cases where one of the tests under investigation involves a high burden with direct side effects for the patient, such as severe bleeding or a high radiation exposure, the classical strategy design should be preferred over the remaining designs, since it is ethically not justified to perform both tests in all patients. An example for a scenario where the test has a direct positive effect on patients is the study by Dreyer et al. (2017) [42] demonstrating a benefit of an oil-based rather than water-based contrast for tubal patency testing in infertile women. However, this design tends to be less efficient, as in many patients tests would yield the same test results, consequently leading to an identical patient management. Hence, if feasible and ethically justifiable, designs evaluating both tests in each patient might be more favorable. Especially, the discordance design, where randomization is restricted to a subgroup of patients with discordant test results, might achieve higher statistical power than the classical RCT design [9, 10]. The design with the random disclosure principle combines the advantages of both, as it can be analyzed like the classical RCT and, additionally, allows subsequent analyses in discordant pairs.

In each design it is assumed that an already existing test *B* is to be replaced by a new test *A* to guide better treatment decisions. Hence, the focus is on the comparison of test-treatment strategies. This requires a well-defined study protocol that describes the specific link between the tests, their results and subsequent management decisions [3]. This may be a direct link such that test-positive patients receive treatment I and test-negative treatment II. However, if in clinical practice decisions are based on more complex processes (including shared decision making in multi-disciplinary clinical teams), the link has to be defined in a less strict manner with obvious challenges for generalizability and replicability of study findings [2, 9]. In case of negative study results, it remains unclear whether it is due to incorrect management decisions, insufficient accuracy of diagnostic testing or ineffective treatments [2, 9]. Unblinding of the clinician and patients included in the trial regarding test results and treatment allocation is an important issue in randomized diagnostic trials, but difficult to implement. Patients’ willingness to undergo testing procedures, especially in case of multiple testing, has to be assured. Knowledge about their test results can influence patients’ further motivation to adhere to treatment and attend follow-up and thus future health outcome is affected [2]. In the context of this work, one primary endpoint is considered to reflect the patient benefit from the test-treatment strategies. In clinical practice, however, there are situations in which the examination of one primary endpoint is not sufficient. In particular, it is important to consider what impact FP and FN test findings may have on the clinical outcome of the patient. One example scenario would be the choice between a curative and a palliative treatment strategy in cancer patients. The consequences of FP or FN test results may be quite different and therefore may not be measurable in the same outcome. A correct decision for curative treatment can lead to improved patient survival, while a correct decision for palliative treatment ideally improves quality of life. From a statistical and technical point of view, there are ways to include more than one primary endpoint in the final analysis. One possibility is to construct a composite endpoint that combines, for example, an improvement in quality of life with an increased survival probability. Other alternatives would be to order the endpoints hierarchically or to consider them with appropriate adjustment of the type I error due to multiple testing. Further possibilities may arise if a reference standard has also been measured in the trial. From an ethical point of view, however, the challenge remains that the potential benefits from increasing the number of TP and TN tested patients cannot be maximized at the same time, especially when tests show a high FP or FN rate. In the planning stage of the study the sample size should be adequately planned to avoid misleading conclusions from the study results. Due to uncertain assumptions regarding involved parameters the calculated sample size may be too small or too large. An adaptation regarding the initial sample size by means of predetermined interim analyses would be desirable to achieve more reliable study results. It would provide information whether to stop recruitment for futility or efficacy or to adjust the sample size. Since research on adaptive designs within randomized test-treatment studies is limited so far, further research is recommended.

## Supplementary Information

**Additional file 1** The Appendix provides formulas to calculate the sample size of a binary and continuous outcome. (PDF 150 KB)

## Data Availability

Not applicable. Declarations

## Abbreviations

RCT: Randomized controlled trial
PEG: Percutaneous endoscopic gastrostomy
MBS: Modified barium swallow test
FEESST: Flexible endoscopic evaluation of swallowing with sensory testing
US: Ultrasonsography
IUGR: Intrauterine growth retardation
HYFoSy: Hysterosalpingo-foam sonography
HSG: Hysterosalpingography
HIV: Human immunodeficiency virus
FP: False positive
TN: True negative
FN: False negative
TP: True negative

## References

[1] Schünemann HJ, Oxman AD, Brozek J, Glasziou P, Jaeschke R, Vist GE, Williams JW, Kunz R, Craig J, Montori VM (2008). Grading quality of evidence and strength of recommendations for diagnostic tests and strategies. BMJ.

[2] di Ruffano LF, Hyde CJ, McCaffery KJ, Bossuyt PMM, Deeks JJ (2012). Assessing the value of diagnostic tests: a framework for designing and evaluating trials. BMJ.

[3] Lijmer JG, Leeflang M, Bossuyt PMM (2009). Proposals for a phased evaluation of medical tests. Med Decis Making.

[4] Lord SJ, Irwig L, Simes RJ (2006). When is measuring sensitivity and specificity sufficient to evaluate a diagnostic test, and when do we need randomized trials?. Ann Intern Med.

[5] Sackett DL, Haynes RB (2002). Evidence base of clinical diagnosis: The architecture of diagnostic research. BMJ.

[6] di Ruffano LF, Dinnes J, Taylor-Phillips S, Davenport C, Hyde C, Deeks JJ (2017). Research waste in diagnostic trials: a methods review evaluating the reporting of test-treatment interventions. BMC Med Res Methodol.

[7] di Ruffano LF, Dinnes J, Sitch AJ, Hyde C, Deeks JJ (2017). Test-treatment RCTs are susceptible to bias: a review of the methodological quality of randomized trials that evaluate diagnostic tests. BMC Med Res Methodol.

[8] Lijmer JG, Bossuyt PMM (2009). Various randomized designs can be used to evaluate medical tests. J Clin Epidemiol.

[9] Lijmer JG, Bossuyt PMM, Knottnerus JA, Buntinx F (2009). Diagnostic testing and prognosis: the randomized controlled trial in test evaluation research. The Evidence Base of Clinical Diagnosis.

[10] Lu B, Gatsonis C (2013). Efficiency of study designs in diagnostic randomized clinical trials. Stat Med.

[11] Vach W, Reiser V, Kolankowska I, Weber S, Rücker G. Design and evaluation of diagnostic studies. University Medical Center Freiburg. 2017. https://www.wb.uni-freiburg.de/inhalte/pdfs/oh-projekt/mfbv/book-diagnosticstudies_mfbv_ruecker_2017. Accessed 12 May 2021.

[12] Bossuyt PMM, Lijmer JG, Mol BWJ (2000). Randomised comparisons of medical tests: sometimes invalid, not always efficient. The Lancet.

[13] Lee CK, Lord SJ, Coates AS, Simes RJ (2009). Molecular biomarkers to individualise treatment: assessing the evidence. MJA.

[14] Freidlin B, McShane LM, Korn EL (2010). Randomized clinical trials with biomarkers: design issues. JNCI.

[15] Young KY, Laird A, Zhou XH (2010). The efficiency of clinical trial designs for predictive biomarker validation. Clin Trials.

[16] Eng KH (2014). Randomized reverse marker strategy design for prospective biomarker validation. Stat Med.

[17] Simon R (2010). Clinical trial designs for evaluating the medical utility of prognostic and predictive biomarkers in oncology. Personalized Med.

[18] Atkinson Jr AJ, Colburn WA, DeGruttola VG, DeMets DL, Downing GJ, Hoth DF, Oates JA, Peck CC, Schooley RT, Biomarkers Definitions Working Group (2001). Biomarkers and surrogate endpoints: preferred definitions and conceptual framework. Clin Pharmacol Ther.

[19] Ziegler A, Koch A, Krockenberger K, Großhennig A (2012). Personalized medicine using DNA biomarkers: a review. Hum Genet.

[20] Buyse M, Michiels S, Sargent DJ, Grothey A, Matheson A, De Gramont A (2011). Integrating biomarkers in clinical trials. Expert Rev Mol Diagn.

[21] Tajik P, Zwinderman AH, Mol BW, Bossuyt PMM (2013). Trial designs for personalizing cancer care: a systematic review and classification. Clin Cancer Res.

[22] Vach W, Høilund-Carlsen PF, Gerke O, Weber WA (2011). Generating evidence for clinical benefit of PET/CT in diagnosing cancer patients. J Nucl Med.

[23] Sargent DJ, Conley BA, Allegra C, Collette L (2005). Clinical trial designs for predictive marker validation in cancer treatment trials. J Clin Oncol.

[24] Aviv JE (2000). Prospective, randomized outcome study of endoscopy versus modified barium swallow in patients with dysphagia. The Laryngoscope.

[25] Gerke O, Vach W, Høilund-Carlsen PF (2008). PET/CT in cancer. Methods Inf Med.

[26] van Rijswijk J, van Welie N, Dreyer K, van Hooff MHA, de Bruin JP, Verhoeve HR, Mol F, Kleiman-Broeze KA, Traas MAF, Muijsers GJJM (2018). The FOAM study: is Hysterosalpingo foam sonography (HyFoSy) a cost-effective alternative for hysterosalpingography (HSG) in assessing tubal patency in subfertile women? Study protocol for a randomized controlled trial. BMC Womens Health.

[27] Nienhuis SJ, Vles JSH, Gerver WJM, Hoogland HJ (1997). Doppler ultrasonography in suspected intrauterine growth retardation: a randomized clinical trial. Ultrasound Obstet Gynecol.

[28] Pepe MS (2003). The Statistical Evaluation of Medical Tests for Classification and Prediction.

[29] Gerke O, Høilund-Carlsen PF, Vach W (2015). Analyzing paired diagnostic studies by estimating the expected benefit. Biom J.

[30] Branscum AJ, Johnson WO, Gardner IA (2007). Sample size calculations for studies designed to evaluate diagnostic test accuracy. J Agric Biol Environ Stat.

[31] Georgiadis MP, Johnson WO, Gardner IA, Singh R (2003). Correlation-adjusted estimation of sensitivity and specificity of two diagnostic tests. J R Stat Soc: Ser C: Appl Stat.

[32] McCray GPJ, Titman AC, Ghaneh P, Lancaster GA (2017). Sample size re-estimation in paired comparative diagnostic accuracy studies with a binary response. BMC Med Res Methodol.

[33] US Food and Drug Administration, et al.Adaptive designs for medical device clinical studies: Guidance for industry and food and drug administration staff. Silver Spring, MD. 2016. https://www.fda.gov/regulatory-information/search-fda-guidance-documents/adaptive-designs-medical-device-clinical-studies. Accessed 12 May 2021.

[34] Zapf A, Stark M, Gerke O, Ehret C, Benda N, Bossuyt PMM, Deeks J, Reitsma J, Alonzo T, Friede T (2020). Adaptive trial designs in diagnostic accuracy research. Stat Med.

[35] Heckman-Stoddard BM, Smith JJ. Precision medicine clinical trials: defining new treatment strategies. In: Seminars in Oncology Nursing: 2014. p. 109–16. 10.1016/j.soncn.2014.03.004 PMC4280791 24794084

[36] Allison M (2010). Biomarker-led adaptive trial blazes a trail in breast cancer. Nat Biotechnol.

[37] Corey L, Nabel GJ, Dieffenbach C, Gilbert P, Haynes BF, Johnston M, Kublin J, Lane HC, Pantaleo G, Picker LJ (2011). HIV-1 vaccines and adaptive trial designs. Sci Transl Med.

[38] Zhang Y, Trippa L, Parmigiani G (2016). Optimal Bayesian adaptive trials when treatment efficacy depends on biomarkers. Biometrics.

[39] Antoniou M, Jorgensen AL, Kolamunnage-Dona R (2016). Biomarker-guided adaptive trial designs in phase II and phase III: a methodological review. PLoS ONE.

[40] Placzek M, Friede T (2018). Clinical trials with nested subgroups: analysis, sample size determination and internal pilot studies. Stat Methods Med Res.

[41] Gao Z, Roy A, Tan M (2016). A two-stage adaptive targeted clinical trial design for biomarker performance-based sample size re-estimation. Stat Biosci.

[42] Dreyer K, van Rijswijk J, Mijatovic V, Goddijn M, Verhoeve HR, van Rooij IAJ, Hoek A, Bourdrez P, Nap AW, Rijnsaardt-Lukassen HGM, Timmerman CCM, Kaplan M, Hooker AB, Gijsen AP, van Golde R, van Heteren CF, Sluijmer AV, de Bruin J-P, Smeenk JMJ, de Boer JAM, Scheenjes E, Duijn AEJ, Mozes A, Pelinck MJ, Traas MAF, van Hooff MHA, van Unnik GA, de Koning CH, van Geloven N, Twisk JWR, Hompes PGA, Mol BWJ (2017). Oil-based or water-based contrast for hysterosalpingography in infertile women. N Engl J Med.

