# Supplementary material for: Randomized test-treatment studies with an outlook on adaptive designs
Source: BMC Med Res Methodol. 2021 Jun 1;21:110. doi: 10.1186/s12874-021-01293-y (PMC8167391; doi:10.1186/s12874-021-01293-y)
Supplement: Supplementary file 1 — Additional file 1 The Appendix provides formulas to calculate the sample size of a binary and continuous outcome. (PDF 150 KB) [file 12874_2021_1293_MOESM1_ESM.pdf]

RESEARCH

# Randomized Test-Treatment Studies with an Outlook on Adaptive Designs

## Additional File 1

Amra Hot[1*], Patrick M. Bossuyt[2], Oke Gerke[3,4], Simone Wahl[5], Werner Vach[6,7†] and Antonia Zapf[1†]

*Correspondence: a.hot@uke.de
[1]Institute of Medical Biometry and Epidemiology, University Medical Center Hamburg-Eppendorf, Martinistraße 52, 20246 Hamburg, Germany
Full list of author information is available at the end of the article
†Shared last authorship

## Appendix

### Sample size formula for a binary outcome

If there is a binary outcome Y of primary interest, the sample size calculation involves a comparison of two binomial proportions $\theta_A$ and $\theta_B$ based on independent samples. Let $\alpha$ denote the type I error probability and $\beta$ the type II error probability. Accordingly, $z_{1-\alpha}$ and $z_{1-\beta}$ are the critical values of the standard normal distribution with upper tail probability of $(1-\alpha)$ and $(1-\beta)$, respectively. We are using a two-sided test to level $\alpha$ testing the hypotheses $H_0 : \Delta = 0$ vs. $H_1 : \Delta \neq 0$, where $\Delta = \theta_A - \theta_B$. Therefore, the sample size formula for testing the inequality of two binomial proportions $\theta_A$ and $\theta_B$ with equal allocation of patients to test $A$ or $B$ can be performed along the following lines:

$$n_A = n_B = \frac{[\sqrt{2\bar{\theta}(1-\bar{\theta})}z_{1-\alpha/2} + \sqrt{\theta_A(1-\theta_A)+\theta_B(1-\theta_B)}z_{1-\beta}]^2}{\Delta^2} \tag{1}$$

where $\bar{\theta} = (\theta_A + \theta_B)/2$ [1]. The number of patients randomized to path $A$ and $B$ is denoted by $n_A$ and $n_B$, respectively.

### Sample size formula for a continuous outcome

Assuming a continuous outcome Y, the primary hypothesis of interest is the mean comparison between two test-treatment strategies based on test $A$ and $B$. Accordingly to the binomial scenario, the difference in mean responses is $\Delta = \theta_A - \theta_B$, where the hypothesis of interest is written as $H_0 : \Delta = 0$ vs. $H_1 : \Delta \neq 0$. If a normal approximation is used and a constant variance for all patients, $\sigma^2$, is assumed, the sample size formula for equal allocation of independent samples and testing inequality of two mean responses is given as [1]:

$$n_A = n_B = \frac{2(z_{1-\alpha/2} + z_{1-\beta})^2\sigma^2}{\Delta^2} \tag{2}$$

Here, $\alpha$ denotes the type I error probability and $\beta$ the type II error probability. Accordingly, $z_{1-\alpha}$ and $z_{1-\beta}$ are the critical values of the standard normal distribution with upper tail probability of $1-\alpha$ and $1-\beta$, respectively. The same applies to the remaining designs.

**Author details**
[1]Institute of Medical Biometry and Epidemiology, University Medical Center Hamburg-Eppendorf, Martinistraße 52, 20246 Hamburg, Germany. [2]Department of Epidemiology and Data Science, Amsterdam University Medical Centers, Meibergdreef 9, 1105 AZ Amsterdam, The Netherlands. [3]Department of Nuclear Medicine, Odense University Hospital, J.B. Winsløws Vej 4, 5000 Odense C, Denmark. [4]Department of Clinical Research, University of Southern Denmark, Winsløwparken 19, 5000 Odense C, Denmark. [5]Roche Diagnostics GmbH, Nonnenwald 2, 82377 Penzberg, Germany. [6]Basel Academy for Quality and Research in Medicine, Steinenring 6, 4051 Basel, Switzerland. [7]Department of Environmental Science, University of Basel, Spalenring 145, 4055 Basel, Switzerland.

**References**
1. Chow SC, Wang H, Shao J. Sample size calculations in clinical research. Florida: CRC press; 2007.
